# Investigating morphological and biological reproductive traits in self-fertile and -infertile macadamia cultivars

**DOI:** 10.1186/s12870-025-07976-8

**Published:** 2026-01-08

**Authors:** Palakdeep Kaur, Max Cowan, Ky Mathews, Mobashwer Alam, Bruce Topp

**Affiliations:** 1 Queensland Alliance for Agriculture and Food Innovation, The University of Queensland, St. Lucia, QLD 4072 Australia; 2 Department of Primary Industries, Maroochy Research Facility, Nambour, QLD 4560 Australia; 3 47 Mayers Road, Nambour, QLD 4560 Australia

**Keywords:** Macadamia, Self-fertile, Self-infertile, Pollen tube growth, Self-incompatibility

## Abstract

Self-fertility is a commercially valuable trait in crop species, enabling fruit set without reliance on pollinators or external pollinisers. In macadamia, most cultivars are self-infertile, though some can produce nuts with self-pollen. The mechanisms underlying this variation remain unclear. This study investigates herkogamy, dichogamy and in vivo pollen tube growth to investigate self-fertility. The traits were measured on cultivars from self-fertile and self-infertile groups. Herkogamy was assessed by pistil length (PL), stamen length (SmL), and stigma-anther distance (SAD). Significant interactions were observed between fertility groups and cultivars (nested within groups), with PL ranging from 11.0 to 14.5 mm, SmL from 6.5 to 9.0 mm, and SAD from 6.6 to 7.9 mm. The non-significant differences in SAD, together with the presence of approach herkogamy (stigma positioned above anthers) in both the self-fertile and self-infertile groups, demonstrated that spatial separation does not explain fertility differences. Temporal separation was assessed via pollen viability and stigma receptivity across six floral stages, where all cultivars exhibited protandry (dichogamy). The overlap in male and female reproductive maturity in all cultivars confirmed that temporal differences do not account for self-fertility. Fluorescence microscopy revealed inhibited pollen tube growth within styles of incompatible pollinations, indicated stylar self-incompatibility. Pollen tube progression ranged from 0 to 73.3% pistils where pollen tube reached the lower style, with significant general, specific, and reciprocal effects. We concluded that self-fertility in macadamia is not governed by spatial or temporal reproductive differences, but instead reflects a homomorphic stylar self-incompatibility mechanism, providing a foundation for future molecular and genetic investigations.

## Background

Macadamia (*Macadamia* spp., Proteaceae) is a subtropical nut tree crop which includes four species— *M. integrifolia* Maiden and Betche, *M. tetraphylla* L.A.S. Johnson, *M. ternifolia* F. Muell and *M. jansenii* C.L. Gross and P.H. Weston (Mai et al. 2020). It is a mass-flowering, partially self-incompatible species (Trueman et al. 2022; Urata 1954), where cross-pollination is essential for achieving optimal nut set and yield (De Silva et al. 2024; Howlett et al. 2019; Trueman et al. 2022; Wallace et al. 1996). Despite producing hermaphroditic florets with both stamens and pistils (Kaur et al. 2024), most commercial cultivars are self-infertile, with only a few capable of setting nuts with self-pollen (Howell et al. 2018; Kaur et al. 2024; Trueman et al. 2024).

Macadamia flowers are not adapted for wind pollination due to the release of pollen in viscous clumps and the sticky nature of the pollen grains (Howlett et al. 2015). Pollination is primarily insect-mediated, with European honeybees (*Apis mellifera*) and native stingless bees (*Tetragonula* spp.) being the dominant pollinators in Australian orchards (Howlett et al. 2015). Effective pollination requires approximately 150 bee visits per raceme (Heard 1993). Studies have consistently shown that supplementary cross-pollination, when compared to open-pollination, significantly enhances reproductive success. For example, Trueman et al. (2022) reported increases of up to 97% in nut-in-shell yield and 109% in kernel yield relative to open-pollinated racemes. Howlett et al. (2019) reported significantly higher nut set in hand cross-pollinated racemes compared to open-pollinated ones, final nut set increases ranging from 1.5% to 55% which varied between cultivars and depends upon pollen donors. Grass et al. (2018) further showed that supplemental hand cross-pollination increased initial and final nut set by 66% and 44%, respectively. These findings highlight the prevalence of cross-pollination limitation in orchards.

Macadamia florets are structurally capable of self-pollination, as self-pollen deposition occurs at the looping stage when stigmas are in close proximity to anthers (Howlett et al. 2015; Trueman 2013). The sticky nature of pollen facilitates adhesion to stigmas (Sedgley et al. 1985), and previous studies have found no significant difference in nut set between autonomous and hand-mediated self-pollination (Howlett et al. 2019; Kaur et al. 2024; Wallace et al. 1996). Sedgley (1983) in pollen tube growth experiments, also found that both bagged-only and hand self-pollinated treatments produced similar results, with all cultivars exhibiting some degree of self-incompatibility. These observations indicate that self-pollen deposition is not inherently limiting, but its effectiveness may depend on genetic compatibility.

Macadamia exhibits protandry, with pollen release preceding stigma receptivity, which typically begins two days after anthesis (Sedgley et al. 1985). Genotypic variation in pistil receptivity and effective pollination period (EPP) has been reported (Meyers 1998), and synchronisation between pollen viability and stigma receptivity is critical for successful self-fertilisation (Kalisz et al. 2012). Despite over a century of macadamia cultivation (Hardner 2016), the mechanisms underlying self-fertility remain poorly understood. Outcrossing plant species typically exhibit greater spatial (herkogamy) and temporal (dichogamy) separation of reproductive organs compared to selfing species (Chabert and Mallinger 2025; Li et al. 2013; Roldán and Ashworth 2018). In other plant species, such as *Leptosiphon jepsonii* (Polemoniaceae) (Goodwillie and Ness 2005) and *Datura stramonium* (Solanaceae) (Motten and Stone 2000), high self-fertility has been associated with reduced spatial separation of reproductive organs. Dichogamy has been shown to prevent selfing in several crops, including *Juglans regia* L. (Juglandaceae) (Kumar et al. 2024), *Castanea* spp. (Fagaceae) (Pauly et al. 2023), *Persea americana* Mill. (Lauraceae) (Alcaraz and Hormaza 2024), *Bridelia* spp. (Phyllanthaceae) (Dias and Ratnayake 2021) and *Narcissus broussonetii* (Amaryllidaceae) (Barranco et al. 2019). But role of herkogamy and dichogamy in self-fertility remains unexplored in macadamia.

Self-incompatibility (SI) is a genetically controlled mechanism that prevents or preventing or inhibits self-fertilisation in hermaphroditic species, involving self/non-self-recognition between pollen and pistil. Multiple SI mechanisms exist, governed by distinct genetic, floral morphological and physiological processes, including gametophytic, sporophytic, homomorphic, heteromorphic, and late-acting SI systems (Chabert and Mallinger 2025; Peer and Mir 2025). Detailed cytological examinations of pollen tube growth is vital for differentiating modes of SI e.g., Millner et al. (2015). For example, in homomorphic sporophytic SI (SSI), self-pollen tubes are inhibited at the stigmatic surface (Nasrallah and Nasrallah 1993); in gametophytic SI (GSI), inhibition occurs within the style (Newbigin et al. 1993); in late-acting SI (LSI), self-pollen tubes reach the ovary but either fertilization fails to occur or fruits/nuts do not reach maturity after fertilisation (Gibbs 2014).

Previous research suggests that macadamia exhibits stylar SI (Sedgley 1983), which is characteristic of GSI (Caruso et al. 2012; Nettancourt 1997; Franklin-Tong and Franklin 2003), although definitive evidence of GSI has not been established. Additionally, LSI, has been observed, with fruitlet abscission occurring 10–13 weeks after self-pollination (De Silva et al. 2024; Meyers 1998). This study investigates the reproductive mechanisms associated with self-fertility in macadamia. Specifically, it examines the spatial separation of reproductive organs (herkogamy), the timing of pollen release and stigma receptivity (dichogamy), and the cytological patterns of pollen tube growth in self-fertile and self-infertile cultivars. Together, these experiments aim to elucidate the biological basis of self-fertility and provide a foundation for future genetic and molecular research in macadamia breeding and orchard management.

## Results and discussion

### Spatial separation of reproductive organs: herkogamy

**Fig. 1:**
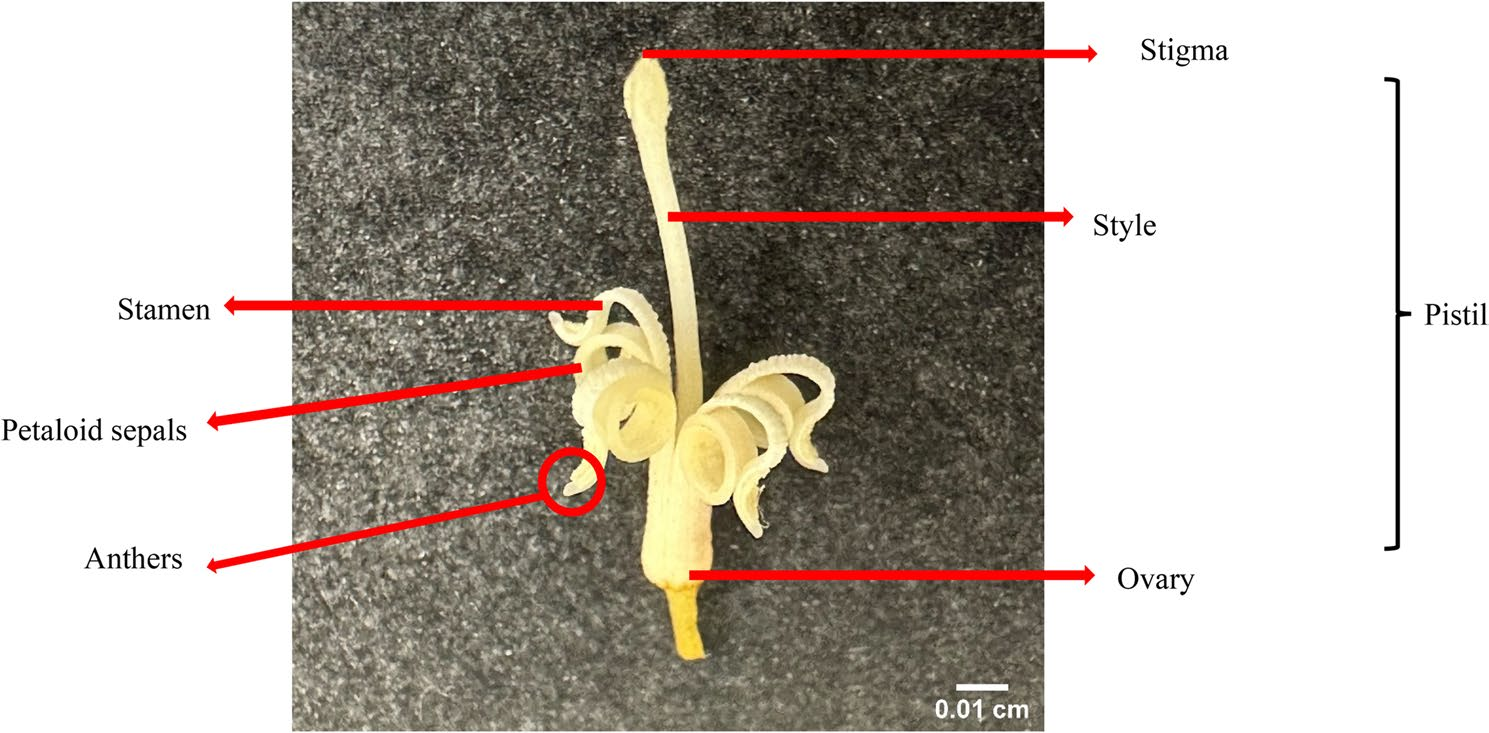
Incomplete and perfect macadamia floret at anthesis stage. It consists of both male (stamen) and female (pistil) reproductive whorls. The non-reproductive whorls - calyx and corolla, are fused to form petaloid sepals. The pistil consists of stigma (apical pollen receptive surface), style (elongated tube connecting stigma to the lower part of the pistil) and ovary (basal region containing ovules). Bar = 0.01 cm

To investigate the role of herkogamy in macadamia self-fertility, floral reproductive morphology was examined across self-fertile and self-infertile cultivars over two flowering seasons (2022 and 2023). All sampled florets were bisexual, containing both stamens and pistils, but morphologically incomplete, lacking distinct petals and sepals, instead presenting four petaloid sepals. Stamens were consistently curled backwards while remaining attached to the petaloid sepals (Fig. 1).

**Fig. 2:**
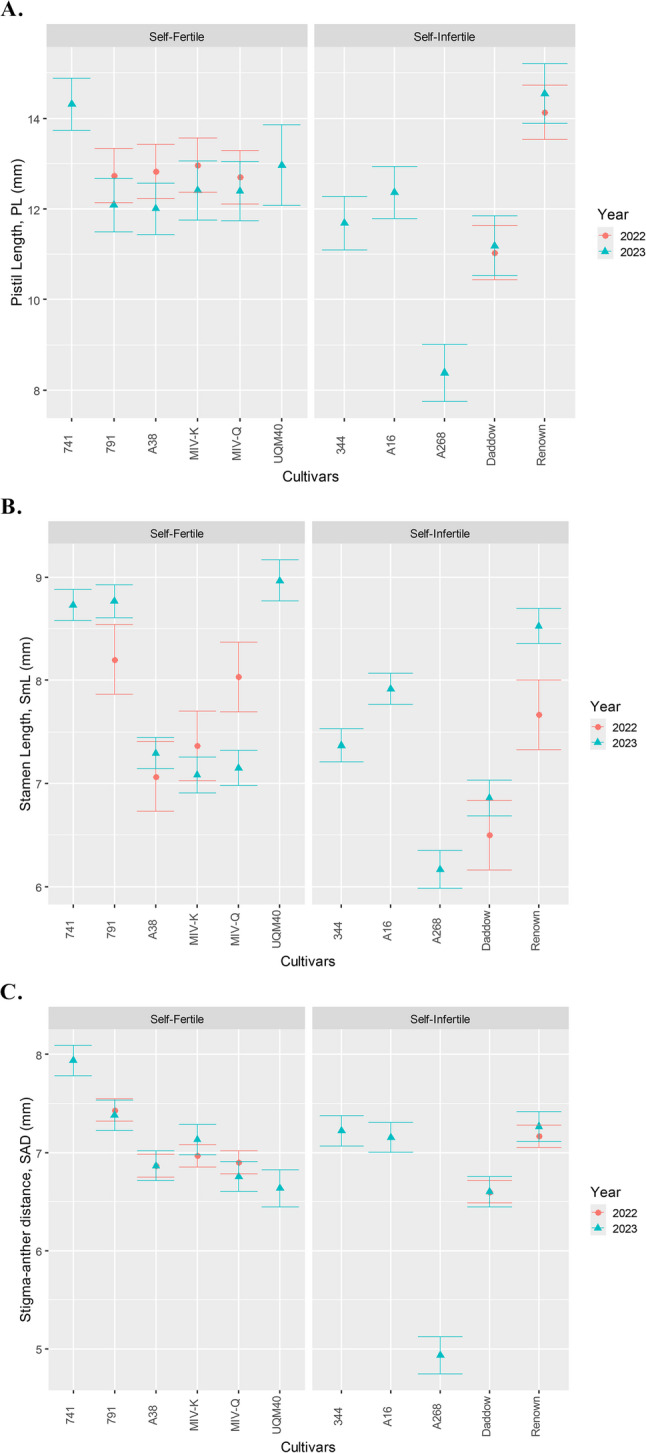
Bars representing mean values of (**A**) pistil length, PL (mm), (**B**) stamen length, SmL (mm), and (**C**) stigma–anther distance, SAD (mm), including standard error bars, for self-fertile and self-infertile macadamia cultivars across two consecutive flowering seasons: 2022 (*n* = 180) and 2023 (*n* = 465)

There was no evidence that the pistil length (*P* = 0.99), stamen length (*P* = 0.06) and stigma-anther distance (*P* = 0.82) is different between self-fertile and self-infertile cultivars across both years (Table 1), indicating trait stability across seasons and cultivar types. This consistency supports the use of these traits in characterising reproductive morphology. However, significant interactions were detected between fertility group and cultivar, and between year and group, suggesting that observed group-level differences may be driven by specific cultivars rather than differences between groups. Notably, the self-infertile cultivar ‘A268’ exhibited markedly lower mean values for PL (8.4 mm), SmL (6.2 mm), and SAD (4.9 mm), compared to other cultivars which ranged from 11.0 to 14.5 mm (PL), 6.5–9.0 mm (SmL), and 6.6–7.9 mm (SAD) (Fig. 2A–C). Additionally, no significant main effect of year was detected, suggesting that reproductive traits are relatively stable across these years, under the environmental conditions observed. The bisexual and incomplete floral morphology observed is consistent with previous findings (du Queens et al. 2018; Urata 1954).

**Table 1:** Wald test statistics for pistil length (PL), stamen length (SmL) and stigma-anther distance (SAD)

Trait	Sources of variation	Degrees of freedom	Wald statistic	Mean squares	*P*-value
Pistil length (PL)	Year	1	0.03	0.03	0.86
	Year: Group	2	18.35	9.17	0.00
	Group: Cultivar	9	243.88	27.1	0.00
	Year: Group: Cultivar	4	0.35	0.09	0.99
Stamen length (SmL)	Year	1	8.93	8.93	0.08
	Year: Group	2	35.59	17.8	0.00
	Group: Cultivar	9	399.54	44.39	0.00
	Year: Group: Cultivar	4	9.48	2.37	0.06
Stigma-anther distance (SAD)	Year	1	0.07	0.07	0.79
	Year: Group	2	18.95	9.48	0.00
	Group: Cultivar	9	194.13	21.57	0.00
	Year: Group: Cultivar	4	1.53	0.38	0.82

**Fig. 3:**
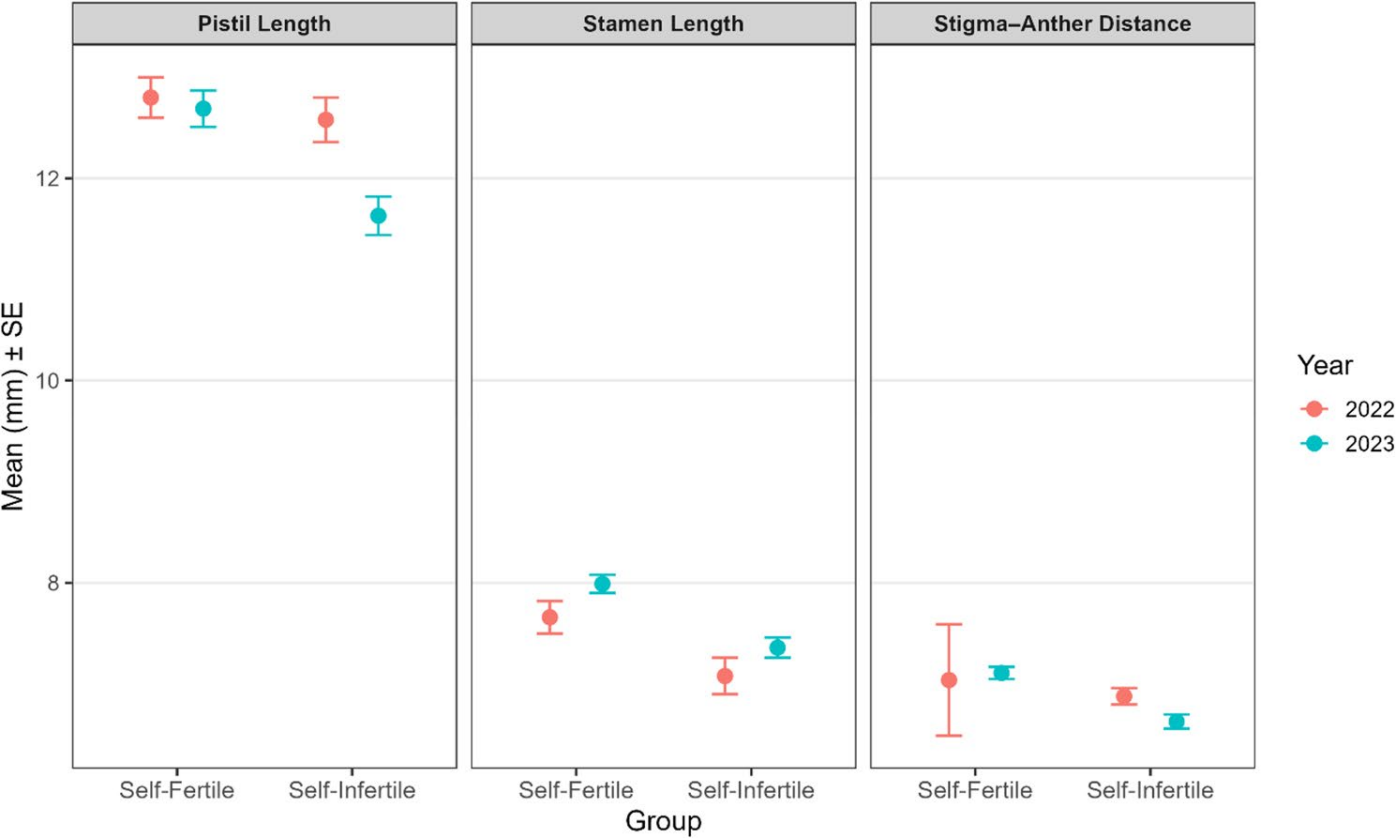
Mean (± standard errors) of pistil length (PL), stamen length (SmL) and stigma-anther distance (SAD) for two years (2022 and 2023)

Reduced stigma-anther separation is generally associated with increased selfing potential due to enhanced contact between self-pollen and the stigma (Fishman et al. 2002). Instead, they showed longer pistils, stamens, and greater stigma-anther distances than self-infertile cultivars (Fig. 3) although non-significant (Table 1), contradicting the hypothesis that minimal spatial separation facilitates self-fertility. The lack of overlapping pistil and stamen lengths further refutes the idea that reduced herkogamy promotes selfing in macadamia.

Despite being morphologically homomorphic and hermaphroditic, macadamia florets consistently displayed approach herkogamy, where stigmas are positioned above the anthers (Opedal 2018). This spatial arrangement is known to reduce sexual interference i.e., unwanted interactions between male and female functions within a flower and is typically associated with promoting outcrossing (Webb and Lloyd 1986). The similarity in floral morphology across self-fertile and self-infertile cultivars suggests that herkogamy alone does not explain differences in self-fertility. Comparable findings have been reported in *Prunus* species, where self-fertility was not linked to floral organ arrangement (De Palma and Godini 1993; Rovira and Miarnau 2016).

### Temporal separation of reproductive organs: dichogamy

In flowering plants, successful fertilisation depends not only on the presence of viable pollen and receptive stigmas but also on the precise timing of their activity. This is especially critical in dichogamous species, where male and female reproductive functions are temporally separated to reduce sexual interference and promote outcrossing (Pant et al. 2020; Wu et al. 2025). Macadamia is known to be protandrous and this study aimed to assess potential differences in the synchronisation of reproductive organs between self-fertile and self-infertile cultivars.

**Fig. 4:**
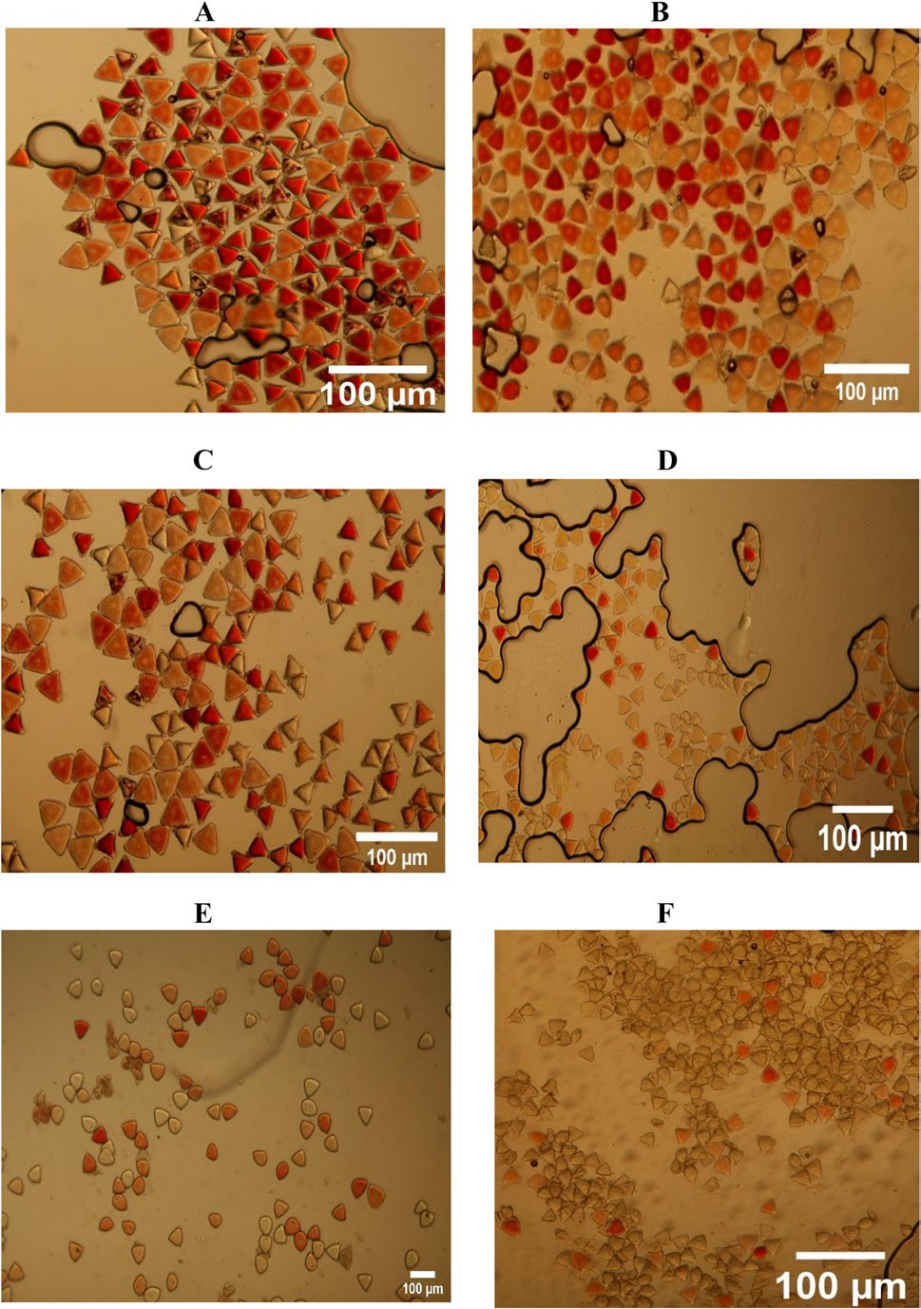
Evaluation of pollen viability of macadamia cultivar ‘791’ using the tetrazolium chloride staining method at six flowering stages (**A**) Looping, (**B**) Anthesis day, (**C**) 1 day after anthesis (DAA), (**D**) 2 DAA, (**E**) 3 DAA, (**F**) 4 DAA. Number of stained pollen grains per 100 pollen grains for each stage were counted to measure the pollen viability. Bar = 100 μm

All cultivars produced morphologically similar pollen, triangular in shape, with no observable differences. No significant differences in pollen viability or stigma receptivity were observed between self-fertile and self-infertile cultivars at any flowering stage (Table 2). Pollen viability peaked at the looping and anthesis stages (91–92% ± 0.37) and declined sharply by 2–4 DAA, reaching minimal levels (2.5–4.5% ± 0.41) (Fig. 4A–F). This rapid decline defines a narrow window for effective pollination.

**Table 2:** Wald statistics table for pollen viability and stigma receptivity

Trait	Flowering stage	Source of variation	Degrees of freedom	Wald statistic	Mean square	*P*-value
**Pollen viability**	Looping	Group	1	1.77	48.39	0.23
		Group: Cultivar	4	8.54	58.23	0.19
	Anthesis	Group	1	0.02	10.97	0.88
		Group: Cultivar	4	0.14	17.45	0.99
	1 DAA	Group	1	0.01	2.81	0.94
		Group: Cultivar	4	0.16	21.46	0.99
	2 DAA	Group	1	0.09	23.51	0.76
		Group: Cultivar	4	0.25	16.08	0.99
	3 DAA	Group	1	0.01	1.08	0.91
		Group: Cultivar	4	6.21	139.44	0.30
	4 DAA	Group	1	3.69	63.85	0.10
		Group: Cultivar	4	10.64	46.02	0.14
**Stigma receptivity**	Looping	Group	1	1.34	2.16	0.29
		Group: Cultivar	4	3.48	1.41	0.53
	Anthesis	Group	1	0.28	0.94	0.62
		Group: Cultivar	4	7.63	6.43	0.23
	1 DAA	Group	1	0.27	0.62	0.62
		Group: Cultivar	4	1.92	1.13	0.75
	2 DAA	Group	1	0.16	0.63	0.70
		Group: Cultivar	4	4.42	4.37	0.43
	3 DAA	Group	1	0.07	0.04	0.79
		Group: Cultivar	4	2.72	0.40	0.63
	4 DAA	Group	1	0.24	0.69	0.64
		Group: Cultivar	4	3.81	2.77	0.49

**Fig. 5:**
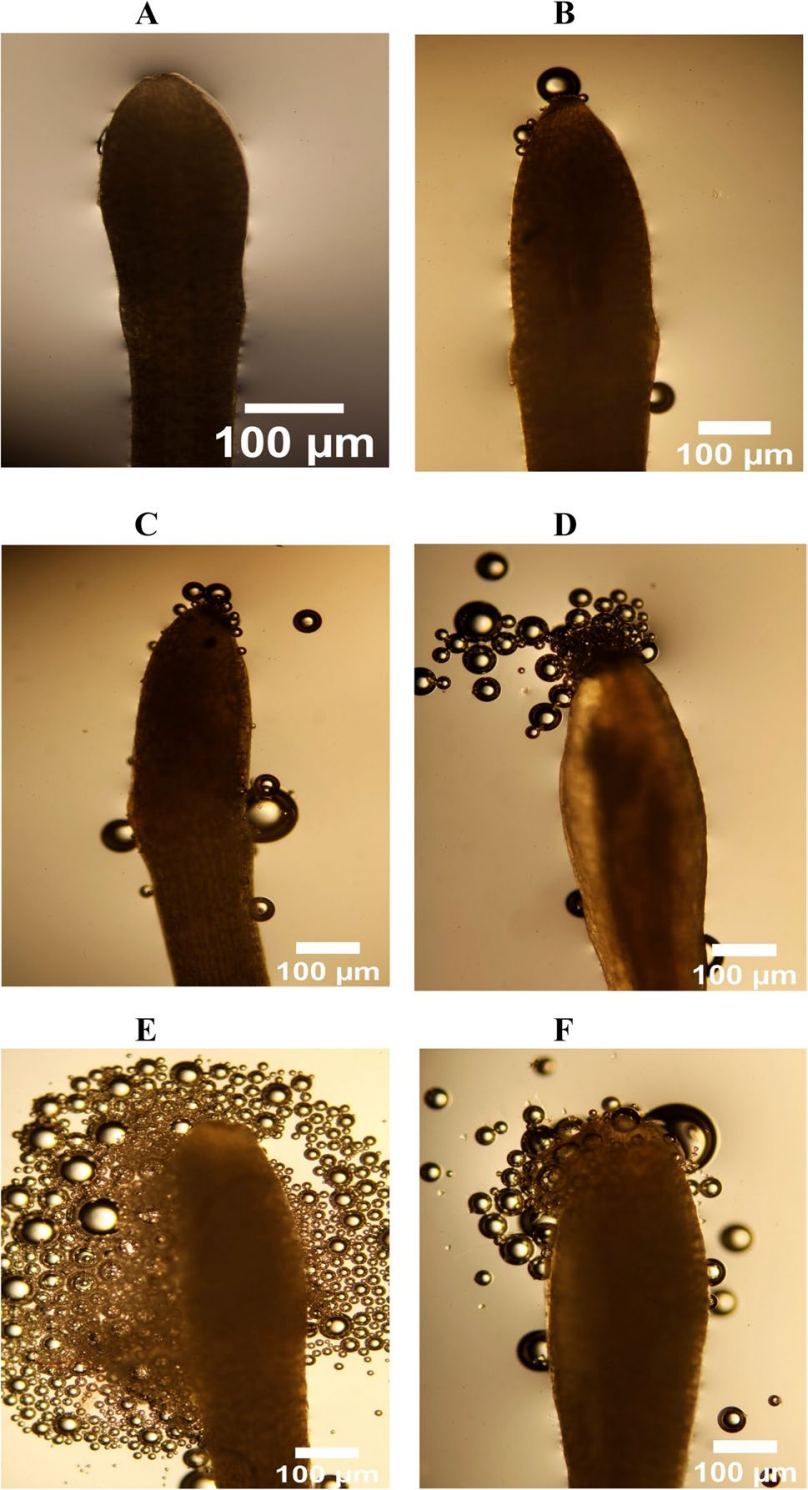
Pollen viability (percentage, solid line) and stigma receptivity (bubbles per minute, dashed line) of self-fertile and self-infertile macadamia cultivars across six flowering stages from looping until 4 days after anthesis. Graphs show mean ± 1 standard error for each cultivar at each flowering stage (*n* = 1800)

**Fig. 6:**
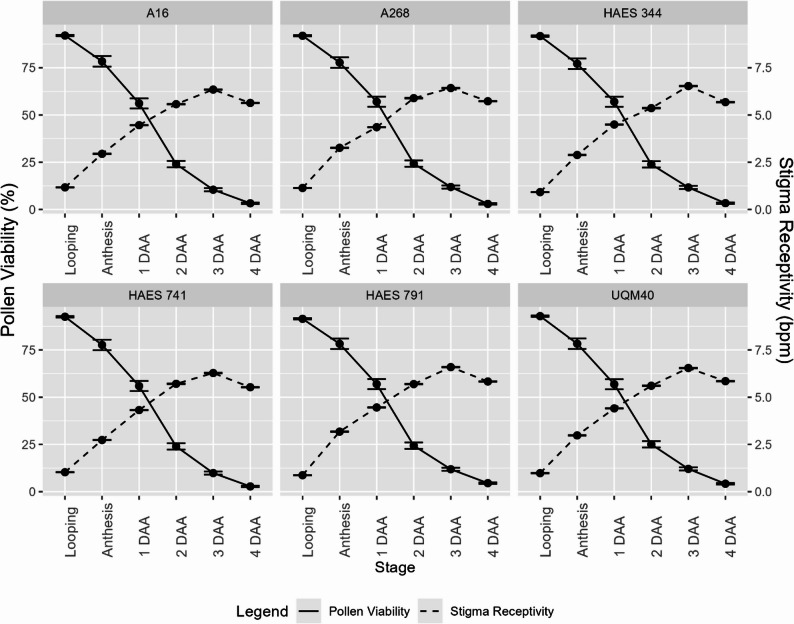
Evaluation of stigma receptivity of macadamia cultivar ‘791’ using hydrogen peroxide (6%) at six flowering stages (**A**) Looping, (**B**) Anthesis day, (**C**) 1 day after anthesis (DAA), (**D**) 2 DAA, (**E**) 3 DAA, (**F**) 4 DAA. Number of bubbles per minute for each stage were counted to test the stigma receptivity. Bar = 100 μm

The stigma is located at the distal, club-shaped end of the style and is lined with papillae. Stigma receptivity is defined as the ability of stigma to permit pollen germination and pollen tube growth, typically associated with elevated esterase and peroxidase activity in mature stigmas (Sharma et al. 2023). Stigma receptivity, assessed via peroxidase activity followed a delayed trajectory (Fig. 5). No bubbling was observed at the looping stage, confirming non-receptivity (Fig. 6A), while peak receptivity occurred at 3 DAA (6.2–7.0 bpm ± 0.16), followed by a slight decline at 4 DAA (5.6 bpm ± 0.14) (Fig. 6E-F).

These patterns of pollen viability and stigma receptivity were consistent across all cultivars (Fig. 5), indicating that protandry is a biological trait in macadamia, regardless of fertility classification. All cultivars exhibited protandry, with viable pollen present at looping and peak stigma receptivity occurring later. These findings suggest that 3 DAA represents the most favourable period for successful pollination, particularly for geitonogamous and cross-pollination events. This uniformity across groups reinforce the conclusion that temporal separation of reproductive organ function (dichogamy) does not explain the observed variation in self-fertility in macadamia.

Similar temporal dynamics have been reported in other species, such as, in *Jatropha curcas* (Euphorbiaceae), pollen viability declines two days post-bloom while stigma receptivity peaks between 1 and 4 days post-bloom (Changwei et al. 2010); in *Melaleuca alternifolia* (Myrtaceae), stigma receptivity initiates on the first day after anthesis, peaks between days three and six, and ceases by day seven (Baskorowati 2009); in *Actinidia deliciosa* (Actinidiaceae), stigma receptivity decreases markedly four days post-anthesis (González et al. 1995). In *Zea mays* (Poaceae), pollen viability declines rapidly by nearly 80% within one hour and completely lost within two hours following anther dehiscence (Luna et al. 2001); in *Hohenbergia amargosensis* (Bromeliaceae), pollen viability drops from 80% to 10% within 12 h post-anthesis (da Silva et al. 2025). These highlight the species-specific nature of reproductive timing and its impact on pollination success.

Anthesis within racemes does not occur synchronously, it may progress acropetally, basipetally, centripetally, or bidirectionally, depending on cultivar and environmental conditions (Trueman 2013). This sequential blooming pattern results in temporal differences of male (pollen release) and female (stigma receptivity) reproductive phases across the raceme, despite individual florets being structurally bisexual. This pattern results in functional dichogamy, where the raceme behaves as functionally unisexual over time. It helps reduce sexual interference i.e., overlap between male and female functions which may hinder fertilisation and affect fruit set (Wu et al. 2025).

At the same time, macadamia exhibits protandry, where male function typically precedes female function within individual florets (Sedgley et al. 1985). However, due to asynchronous anthesis, some florets may simultaneously express male and female functions, a condition known as adichogamy. In adichogamy, reproductive phases are active concurrently but without synchrony across florets, allowing for geitonogamous and cross-pollination. Thus raceme-level timing is crucial for pollination success. Studies in other raceme-bearing species *Aconitum grossedentatum* (Ranunculaceae) show that synchronised dichogamy can reduce geitonogamy and improve seed set by outcrossing (Ida and Minato 2020). These dynamics present avenues for future experimental work to evaluate how raceme-level sexual timing can influence reproductive success.

In current study, florets were selected at similar flowering stages and positioned closely along the raceme to minimise positional variation. Additionally, pollen is sticky and stigmas are moist, which limits passive movement. These traits reduce the likelihood of gravity significantly influencing pollen transfer (Sedgley et al. 1985). Therefore, observed differences in fertility among cultivars are unlikely to be attributed to gravity.

In this study, TTC and H₂O₂ assays were employed which are indirect methods to assess pollen viability and stigma receptivity, respectively. These biochemical tests are widely used in reproductive biology due to their simplicity, rapid execution, and ability to provide preliminary insights into the functional status of reproductive organs (Dafni and Maués 1998; Shivanna and Tandon 2014). In this study, these assays were particularly useful for comparing cultivars from different fertility groups, offering a practical approach to evaluate potential differences in reproductive timing. The consistent results across all cultivars provided confidence that dichogamy was not associated with self-fertility status, at the level detectable by these methods. However, TTC and H₂O₂ assays may not fully capture the physiological or molecular complexity of reproductive compatibility (Shivanna and Tandon 2014). But their use in this study represents a pragmatic and informative first step in characterising reproductive organ activity in macadamia.

### Pollen tube growth

**Fig. 7:**
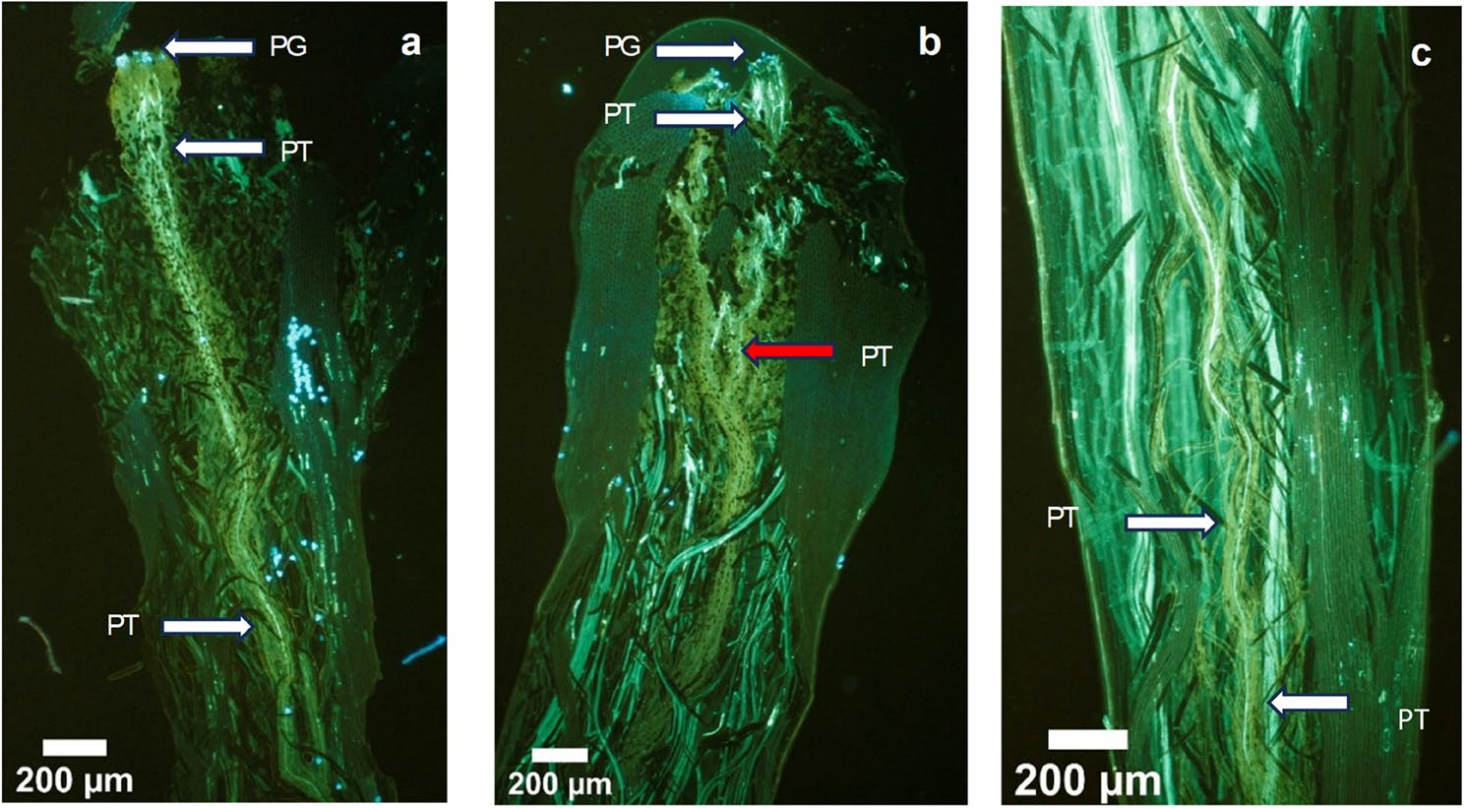
Flourescence microscopy images of in vivo pollen tube growth 7 days after anthesis. (**a**) Pollen grains and pollen tubes on stigmatic surface and within style in completible pollination, (**b**) Pollen grains germinated on stigmatic surface and pollen tube growth inhibited in incompatible pollination, (**c**) Pollen tubes growing in style tissue in compatible pollinations. White arrows indicate bundles of pollen tubes. Red arrow indicates the site of pollen tube inhibition. PG, pollen grains, PT, pollen rube. Bar= 200 μm

Self-infertility poses a significant challenge for orchard design, as successful fruit set relies on the presence of compatible pollinisers and effective pollinator activity. To investigate the physiological basis of self-fertility, this study employed fluorescence microscopy to track pollen tube progression within pistils a reliable and accurate method for characterising self-(in)fertility phenotypes (Millner et al. 2015; Shamsolshoara et al. 2022). In this study, no pollen tubes reached the lower style in self-pollinated pistils of ‘344’ and ‘A16’, confirming their self-infertility. In contrast, pollen tubes successfully reached the lower style in self-pollinated pistils of ‘741’ and ‘791’, indicating self-fertility (Fig. 7).

Meyers (1998) reported fruit retention up to 13 weeks post-pollination in ‘A4’ following both self- and cross-pollination (‘A4 × A16’), describing this phenomenon as proteoid SI, a form of LSI. In the present study, in compatible pollinations, pollen grains germinated on the stigma, and pollen tubes traversed the transmitting tissue to reach the ovary to realize fertilisation. In incompatible pollinations, pollen tube growth was inhibited in the upper third, club-shaped region of the style (Fig. 7). The observed stylar inhibition of pollen tube growth is consistent with the GSI mechanism, wherein self-pollen tubes are arrested within the style, preventing fertilization (Caruso et al. 2012; Sedgley 1983). To confirm the presence and functionality of GSI in macadamia, comprehensive genetic analyses such as diallel crossing experiments, alongside transcriptomic profiling of reproductive tissues, are essential. These approaches can elucidate the molecular basis of self-pollen rejection and identify key regulatory genes involved in the incompatibility response.

**Fig. 8:**
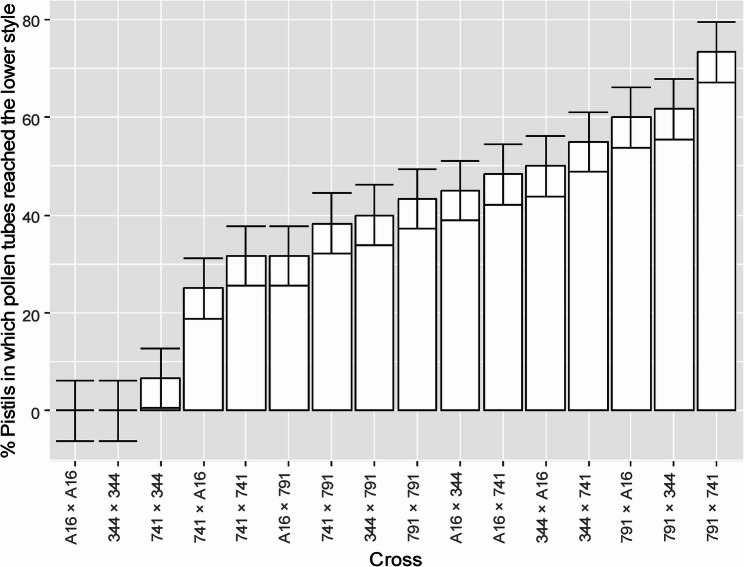
Macadamia crosses in order of increasing compatibility. Bars represent percent of pistils per raceme in which pollen tubes reached the lower style. In each cross, the first parent listed is the female parent. Columns show mean ± standard error bars (sample size per cross = 60; total sample size = 960)

A 4 × 4 full diallel analysis revealed significant variation among crosses (supplementary table S1). The percent of pistils per raceme in which pollen tubes reached the lower style varied from 0.0 to 73.3% (Fig. 8). Among self-pollinations, ‘791’ showed the highest success (43%), followed by ‘741’ (32%). A cross between two self-fertile cultivars ‘791 × 741’ was the most compatible, with 73.3% of pistils per raceme exhibiting pollen tube growth to the lower style. In contrast, ‘741 × 344’ was the least compatible (6.7%) while the reciprocal cross ‘344 × 741’ showed markedly higher compatibility (55%). Interestingly, self-pollination of ‘741’ (32%) resulted in higher pollen tube penetration than some of its cross combinations, such as ‘741 × 344’ (6.7%) and ‘741 × A16’ (25%), suggesting partial cross-incompatibility (CI). Similar patterns have been reported previously, where certain cross combinations failed to outperform self-pollination in fruit set (Meyers 1998). (Meyers 1998). Trueman and Turnbull (1994) also observed poor compatibility in cultivar ‘660’ when pollinated with ‘344’, compared to ‘333’ and ‘246’.

Macadamia cultivars showed significant effects for general compatibility (GC), specific compatibility (SC), and reciprocal effects following self- and cross-pollinations (Table 3). This suggests that cultivar combinations within an orchard can substantially influence yield.

**Table 3:** Sum squares, mean squares and probabilities from compatibility analysis of a 4 × 4 Diallel cross, based on pollen tube growth (percentage of pistils per raceme in which pollen tubes reached the lower style). Effects include general compatibility (GC), specific compatibility (SC), and reciprocal effects

Source of variation	Degrees of freedom	Sum of Squares	Mean squares	*P*-value
General compatibility (GC)	3	51,401	17,134	0.00
Specific compatibility (SC)	6	26,784	4464	0.00
Reciprocal	6	36,633	6105	0.00

**Fig. 9:**
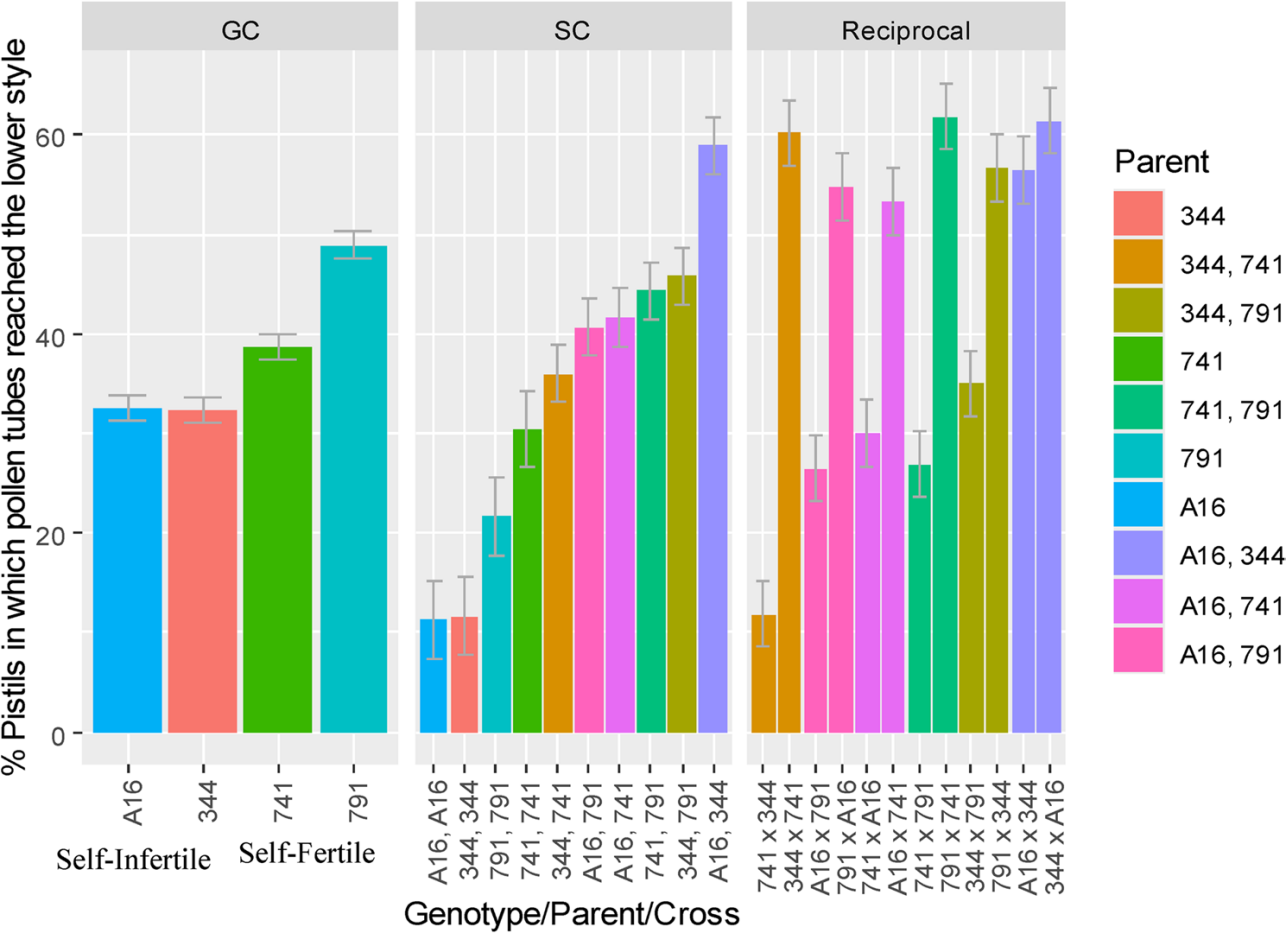
Effects of general compatibility (GC), specific compatibility (SC), and reciprocal crosses in macadamia cultivars, measured as the percentage of pistils in which pollen tubes reached the lower style. Bars represent the standard error of the respective effects based on this response variable. The left panel shows GC effects, the centre panel shows SC effects, and the right panel shows reciprocal effects. In the reciprocal effects panel, two side‑by‑side bars of the same colour represent reciprocal cross combinations, with the first parent in each cross designated as the female parent

The overall GC and SC means were added to their respective effects to obtain means on the measured scale i.e., percent of pistils per raceme in which pollen tubes reached the lower style (Fig. 9). For the reciprocal effects, both the overall mean and SC effect of each cross was included in the respective reciprocal effect. GC effects were lowest for self-infertile cultivars ‘344’ and ‘A16’ (32%), and highest for self-fertile cultivars ‘741’ (38.7%) and ‘791’ (48.9%), with ‘791’ identified as the strongest general combiner due to its high self- and cross-compatibility (Fig. 8). This highlights the importance of the self-fertility trait in macadamia orchards where along with cross-pollinations, self-pollinations have the potential to provide consistent yield. High SC effects were observed in combinations such as ‘A16, 344’; ‘344, 791’; ‘741, 791’, ‘A16, 741’, identifying them as promising specific combiners. Significant reciprocal effects were detected in several crosses, including ‘344 × 741’, ‘791 x A16’, ‘A16 × 741’, ‘791 × 741’ and ‘791 × 344’, suggesting that pollen donor identity can potentially influence fertilisation outcomes (Fig. 9). Notably, no significant difference was observed between reciprocal crosses of ‘A16’ and ‘344’, supporting their high bidirectional compatibility with the highest SC (59.0%).

SC variance analysis (Table 4) further supported these findings where ‘741’ exhibited the lowest SC variance, indicating consistent compatibility across its crosses. While ‘344’ and ‘A16’ showed high SC variance, reflecting uneven compatibility with some combinations where combinations such as ‘A16, 344’; ‘344, 791’; ‘A16, 741’ showed high SC effect, in contrast, ‘A16, A16’; ‘344, 344’ showed low SC effect (Fig. 9). Although, ‘791’ achieved greater self-pollination success, the SC effect was lower than that of ‘741’ self. This discrepancy is attributed to broader variability in compatibility observed across crosses involving ‘791’. In contrast, the consistent compatibility of ‘741’ across its crosses enhanced its SC effect despite lower self-fertility (Fig. 8).

**Table 4:** Estimates of specific compatibility (SC) variances associated with each parent in a 4 × 4 Diallel cross of macadamia cultivars, based on pollen tube growth (percentage of pistils per raceme in which pollen tubes reached the lower style)

Group	Parent	SC variance
Self-Fertile	741	7.22
Self-Fertile	791	31.61
Self-Infertile	A16	205.65
Self-Infertile	344	228.14

These findings underscore the importance of reciprocal effects in orchard design and suggest that planting more than two cultivars may mitigate compatibility-related yield risks. Previous studies have shown that pollen source affects nut set, nut-in-shell mass, kernel mass, and oil concentration (Howlett et al. 2019; Trueman et al. 2022). For example, cultivars ‘741’ and ‘842’ did not show improved nut set when cross-pollinated with ‘344’ compared to other pollen sources (Howlett et al. 2019). An 11% increase in nut mass was observed for ‘246’ when pollinated by ‘A16’ compared to ‘814’ (Herbert et al. 2019). Currently, no universal pollen donor has been identified for macadamia, and cross-compatibility varies significantly between cultivar pairs. This underscores the need for further research to identify optimal polliniser combinations and develop guidelines for orchard design that enhance cross-pollination efficiency.

Despite the value of controlled pollination, manual emasculation and precise pollen brushing are challenging in macadamia due to the small and delicate florets. To facilitate controlled pollination under field conditions, this study employed the hollow tube method (see Materials and Methods). This technique has been previously used in macadamia pollination studies and shown to produce differences in nut set among pollen donors (Howlett et al. 2019; Urata 1954). While this method does not allow for precise quantification of pollen deposition, it offers a practical approach for evaluating compatibility across diverse genotype combinations. In the present study, the cross between a self-infertile mother and a self-fertile pollen donor (‘344 × 741’) resulted in 55% of pistils per raceme showing pollen tube progression to the lower style. In contrast, their reciprocal cross yielded only 6.7%, observed in two pistils sampled from two separate racemes on a single ‘741’ tree. Crosses between two self-infertile cultivars (‘344 × A16’ and ‘A16 × 344’) showed 45%–50% pollen tube penetration, further demonstrating the efficacy of the tube method.

Further, to minimise self-pollen interference, racemes were pre-cleaned by gentle rubbing prior to donor pollen application, and surrounding florets were removed to reduce inter-floral competition. The sticky nature of macadamia stigmas facilitated pollen adhesion, enhancing the likelihood of successful pollen transfer. However, the absence of controlled pollen dosage introduces uncertainty when interpreting pollen tube progression, as variation in pollen production among donor cultivars may influence outcomes. Similar concerns have been raised in other crop systems, where pollen load and deposition uniformity significantly affect fertilisation success and compatibility assessments (Shivanna and Tandon 2014). This highlights the importance of considering additional reproductive traits such as the pollen–ovule ratio (P/O) where high P/O ratios are typically associated with outcrossing, while lower ratios suggest self-compatibility (Burd 2011; Cruden and Miller-Ward 1981). Future studies should aim to quantify pollen deposition per stigma and, track pollen tube growth at multiple stylar levels, integrating P/O ratios to deepen understanding of compatibility mechanisms.

Importantly, the inhibition of pollen tube growth in self-infertile cultivars, despite pollen adhesion, supports that self-fertility in macadamia is governed by a genetically regulated SI system, rather than mechanical barriers such as herkogamy or dichogamy. Self-(in)fertility in many horticultural crops, including apple (Brancher et al. 2021), citrus (Cao et al. 2024), olive (Breton et al. 2016), and sweet cherry (Yildirim and Demirtaş 2021), is typically governed by a single S-locus with multiple alleles. Modifier genes outside the S-locus have also been implicated in the breakdown of SI such as in almonds (Fernández i Martí et al. 2011) and sweet cherry (Ono et al. 2020). Further molecular studies are needed to identify genes associated with self-fertility and determine the underlying regulatory mechanisms.

## Conclusion

This study provides the evidence that self-fertility in macadamia is not governed by floral morphology or reproductive timing. The presence of similar morphology and protandry in self-fertile and self-infertile groups rules out herkogamy and protandry as determinants of self-fertility. Instead, inhibited pollen tube growth within the style in self-incompatible pollinations supports the presence of homomorphic stylar self-incompatibility mechanism. These findings mark a significant step forward in understanding the reproductive biology of macadamia and establish a physiological framework for future molecular investigations. Deciphering the genetic basis of self-fertility will be critical for breeding strategies aimed at enhancing yield stability and reducing pollination dependency in commercial orchards.

## Materials and methods

### Spatial separation of reproductive organs: herkogamy

Reproductive morphological traits were measured during the 2022 and 2023 flowering seasons in cultivars from two groups – self-fertile and self-infertile (Table 5). All trees were located at the Maroochy Research Facility, Queensland Department of Primary Industries, Nambour, Queensland. The cultivars used in this study are either *M. integrifolia* or hybrids of *M. integrifolia* and *M. tetraphylla*. Both fertility groups represent similar composition of species and origin i.e., Hawaiian or Hidden Valley Plantations. Interestingly, cultivars ‘HAES 741’ and ‘HAES 344’ are pure *M. integrifolia* and are closely related, having been derived from the same Hawaiian breeding population (Alam et al. 2018). These cultivars share similar vegetative and floral characteristics such as leaf morphology and inflorescence colour but differ in self-fertility. For clarity, numerical identifiers were assigned to HAES (Hawaiian) cultivars.

**Table 5:** Cultivars and number of trees selected for the herkogamy experiment conducted in 2022 and 2023, and for the dichogamy experiment in 2023. References are provided for species characterisation and for classification of cultivars into self-fertile and self-infertile groups

			Number of trees for Herkogamy		Number of trees for Dichogamy	
**Group**	**Cultivar**	**Species**	**2022**	**2023**	**2023**	**References**
Self-Fertile	HAES 791	Int and Tet	2	3	2	(Kaur et al. 2024; Peace et al. 2002)
	HAES 741	Int	-	3	2	(Kaur et al. 2024; Peace 2005)
	MIV-Q	Int and Tet	2	3		(Howell et al. 2018); (Bruce Topp, Mobashwer Alam, pers. comm.)
	MIV-K	Int	2	3		
	UQM40	Int and Tet	-	2	2	(Peace 2005); (Bruce Topp, Mobashwer Alam, pers. comm.)
	A38	Int	2	3		
Self-Infertile	Daddow	Int	2	3		(Peace 2005; Sedgley 1983)
	Renown	Int and Tet	2	3		(Peace et al. 2002; Sedgley 1983)
	HAES 344	Int	-	3	2	(Peace 2005) (Kaur et al. 2024; Peace et al. 2002)
	A16	Int and Tet	-	3	2	(Peace et al. 2002)
	A268	Int and Tet	-	2	2	(Peace et al. 2002); (Bruce Topp, Mobashwer Alam, pers. comm.)

*Abbreviations*: *A* A-series from Hidden Valley Plantation, *HAES* Hawaiian Agricultural Experiment Station, *MIV* Macadamia Industry Variety from Australian National Breeding Program, *UQM* University of Queensland Macadamia from Australian National Breeding Program, Int, *M. integrifolia*; Tet, *M. tetraphylla*

To quantify herkogamy, three racemes per tree were sampled, with five florets per raceme (15 florets/tree). Florets at anthesis (supplementary figure S1) were collected and transported in moistened containers to prevent desiccation. PL, SmL and SAD were measured using a millimetre-scale ruler (± 0.1 mm) by a single observer to reduce bias; a subset was re-measured to confirm consistency (supplementary figure S2 A- B) as follows:

Pistil Length (PL): Measured from the ovary base to the stigma tip.

Stamen Length (SmL): Measured from the filament base to the anther tip.

Stigma-Anther Distance (SAD): Linear distance between the stigma tip and the nearest anther tip indicating spatial separation of reproductive organs.

### Temporal separation of reproductive organs: dichogamy

Pollen viability and stigma receptivity were evaluated on six cultivars each with two trees in the 2024 flowering season using the same cultivar groups and location as in the herkogamy experiment (Table 5). Racemes were tagged at pre-looping stage and individually enclosed in 0.1 mm mesh bags at looping stage to prevent outcross-pollen contamination (supplementary figure S1). To ensure uniform floral development, open florets were removed prior to bagging, and unopened florets were discarded at anthesis. Pollen grains and stigma were collected from bagged racemes at six flowering stages: looping, anthesis, 1 DAA, 2 DAA, 3 DAA and 4 DAA. For each stage, five racemes per tree were sampled, with five florets per raceme, totalling 1,800 florets for each experiment.

Pollen viability was immediately assessed using 2,3,5-triphenyltetrazolium chloride (TTC) staining (Shivanna and Rangaswamy 2012). TTC penetrates viable pollen, where mitochondrial succinate dehydrogenase reduces it to red formazan, indicating viability. Non-viable pollen remains unstained. Stigma receptivity was evaluated using a 6% hydrogen peroxide (H₂O₂) assay. A drop of H₂O₂ was applied to each stigma, and bubbling was observed under 40× magnification. Effervescence indicates peroxidase activity in receptive stigmas; absence of bubbles denotes non-receptivity. Prior to testing, stigmas were examined for surface damage that could potentially induce enzymatic activity.

### Pollen tube growth

In August-September 2023, a 4 × 4 full diallel cross with controlled self and cross-pollinations was conducted between self-fertile (‘741’, ‘791’) and self-infertile cultivars (‘344’, ‘A16’) at the Maroochy Research Facility, Nambour (supplementary table S2). Two trees per cultivar were selected. For each cross, three racemes per tree were pollinated and ten pistils per raceme were collected, totalling 960 pistils. Racemes were enclosed in 0.1 mm mesh bags at the looping stage to prevent contamination (supplementary figure S1). Floral synchrony was maintained by removing open florets before bagging and discarding unopened florets at anthesis.

Racemes underwent either autonomous self-pollination or were manually cross-pollinated using the hollow tube method (Kaur et al. 2024). Pollen was collected by rubbing the inner surface of a clean plastic tube against freshly opened racemes. Recipient racemes were inserted and rotated within the tube to ensure even pollen deposition. Florets were not emasculated, as attempts to remove anthers prior to looping resulted in stigma desiccation. Consequently, the possibility of self-pollen contamination in a small number of pistils cannot be excluded. Routine genotyping of families produced by tube method indicate zero or very low contamination (Topp and Neal 2015). Racemes were re-bagged post-treatment and monitored for damage. Pistils from cross-pollinated racemes were collected 7 days post-pollination, and from self-pollinated racemes 7 days post-anthesis.

Tissue preparation followed a modified protocol from Shivanna et al. (1992). Samples were fixed in FAA fixative (formalin:10 ml, glacial acetic acid: 5 ml, ethanol:50 ml, distilled water: 35 ml), transferred to 70% ethanol after 24 h fixation and stored at 4^o^ C. Prior to microscopy, pistils were softened in 8 M NaOH for 24 h, rinsed twice with distilled water and stained with 0.1% aniline blue in 0.1 N K_3_PO_4_. After 4–12 h of staining, samples were squashed on slides and examined under an Olympus B071 fluorescence microscope. Aniline blue contains fluorochrome that binds to β−1,3-glucans (callose) in growing pollen tubes, fluorescing at 500/506 nm under UV light (Zavaliev and Epel 2015), allowing visualization of tube progression and inhibition sites.

### Data analysis

All statistical analyses were performed using a linear mixed models in ASReml-R (Butler et al. 2018; VSNiTeam 2023) on the R statistical computing platform (RCoreTeam 2024). Data visualization was performed using the *ggplot2* package (Wickham 2016).

#### Spatial separation of reproductive organs: herkogamy

Year, group (self-fertile and self-infertile), and cultivars (nested within groups) and their interactions were considered as fixed effects. Block, tree, raceme, and floret and their interactions with year were fitted as random effects.

#### Temporal separation of reproductive organs: dichogamy

Pollen viability is defined here as the ability to germinate or set seeds after pollination. For testing, 100 pollen grains per floret were evaluated, and the number of stained pollen grains were counted. Pollen viability (%) was calculated by:

$$\begin{aligned} Pollen\,viability \left(\%\right)=\left(\frac{Number\,of\,stained\,pollen\,grains}{Total\,number\,of\,pollen\,grains\,per\,floret}\right)\times 100\end{aligned}$$

where the total number of pollen grains monitored is 100 for all florets.

Stigma receptivity was calculated by counting the number of bubbles per minute following application of 6% hydrogen peroxide. The higher the number of bubbles per minute, the higher the stigma receptivity. As bubble count data were non-normally distributed—a square root transformation was applied prior to analysis. The trait was recorded as “bubbles per minute (bpm).”

Each flowering stage was analysed independently. Group and cultivars (nested within groups) were fitted as fixed effects, while tree and raceme (nested within tree) were included as random effects. Florets constituted the residual error term. For pollen viability, the tree random effect was unconstrained, resulting in negative variance component estimates for looping, anthesis, 3 DAA and 4 DAA. To avoid singularities due to minimal variability, the tree variance component was fixed at zero for 0 DAA, 1 DAA, and 2 DAA. For stigma receptivity, the tree random effect was also unconstrained, yielding negative variance component estimates across all six flowering stages.

#### Pollen tube growth

Pollen tube progression to the lower style was assessed as a binary trait at the floret level, with values of 0 indicated inhibited and 1 indicated successful pollen tube growth to the lower style. For statistical analysis, floret-level data were aggregated to the raceme level and expressed as a continuous variable: the percentage of pistils per raceme in which pollen tubes reached the lower style. This aggregate trait captures the proportion of successful pollen tube growth events within each raceme and was calculated using the following formula:

$$\begin{aligned} &Pistils\:in\:which\:pollen\:tubes\:reached\:the\:lower\:style\\&=\left(\frac{\varSigma\:\:Pollen\:tubes\:per\:raceme\:per\:cross\:per\:tree}{Number\:of\:pistils\:per\:raceme\:per\:cross\:per\:tree}\right)\times\:100\end{aligned}$$

Given that the number of pistils per raceme per cross per tree was consistently 10, the formula simplifies to:

$$\begin{aligned} &\%\:Pistils\:in\:which\:pollen\:tubes\:reached\:the\:lower\:style\\&=\left(\frac{\varSigma\:Pollen\:tubes\:per\:raceme\:per\:cross\:per\:tree}{10}\right)\times\:100\end{aligned}$$

Diallel cross analysis was conducted using Griffing (1956) Method 1, mixed model A, which partitions the variation into population mean, general combining ability (GCA), specific combining ability (SCA), reciprocal effects and experimental error. GCA reflects the average performance of a genotype across multiple crosses, while SCA refers to the performance of a specific cross (over or under) that would be expected based on the GCA(Henderson 1952). Reciprocal effects represent differences between directional cross (‘A x B’ and ‘B x A’), which are critical for selecting compatible male and female parents. Usually, GCA and SCA are measured on the traits of the progeny. However, in this study, the pollen tube growth was observed to assess compatibility rather than progeny traits. Thus, the terms ‘general compatibility’ (GC) and ‘specific compatibility’ (SC) were adopted to more accurately describe the observed effects. GC represents the average compatibility of a cultivar across all crosses, while SC captures cultivar-specific deviations in pollen tube success. GC, SC and reciprocal effects were estimated as Griffing (1956):

$$\:\begin{array}{c}{gc}_{i}=\frac{1}{2p}\left({X}_{i.\:}+\:{X}_{.i\:}\right)-\frac{1}{{p}^{2}}X..\\\:{sc}_{ij}=\frac{1}{2}\left({x}_{ij\:\:}+{x}_{ji}\:\right)-\frac{1}{2p}\left({X}_{i.\:}+{X}_{.i}+{X}_{j.}+{X}_{.j}\right)+\frac{1}{{p}^{2}}X..\\\:{rc}_{ij}=\frac{1}{2}({x}_{ij}-{x}_{ji})\\\:\mu\:=\frac{1}{{p}^{2}}X..\end{array}$$

where:

$\:{gc}_{i}$ = The general compatibility effect of the *ith* female.

$\:{sc}_{ij}$ = The specific compatibility effect of the cross between *ith* female and *jth* male.

$\:{rc}_{ij}$ = The reciprocal compatibility effect from the crossing between *ith* and *jth*.

$$\:p$$ = Parents.

$\:X..$ = The arithmetic mean of all crosses.

$\:{X}_{i.\:}$ = The arithmetic mean of the crosses where *i* is involved as a parent.

$\:{X}_{j.}$= The arithmetic mean of the crosses where *j* is involved as a parent.

$\:{x}_{ij}$ = Mean value for the F1 resulting from the cross between *ith* female and *jth* male.

$\:\:{x}_{ji}$= Represents the reciprocal of $\:{x}_{ij}$.

$\:\mu\:\:$= The general mean parents or intercept.

The SC effects were compared after computing the SC variance for each parent:

$$\:{\sigma\:s}_{i}^{2}\:\:=\frac{1}{p-2}\sum\:_{j\ne\:i}{sc}_{ij}^{2}-\frac{p-3}{p-2}{\sigma\:}^{2}\:$$

where, $\:{\sigma\:s}_{i}^{2}$ = Specific compatibility variance of the *ith* parent.

$\:{sc}_{ij}^{2}$ = Square of the specific compatibility effect of the cross between *ith* female and *jth* male.

$\:{\sigma\:}^{2}$ = Error variance component.

The treatment structure included two factors, the overall mean and cross. Cross contains 16 levels comprising 4 self-pollinations, 6 direct crosses (C1-C6) and 6 reciprocal crosses (R1-R6) (supplementary table S2). Both mean and cross were fitted as fixed effects. The random effects model accounted for the hierarchical plot structure: individual trees (8 in total) and bags nested within trees (4 bags per tree, 32 in total). Racemes nested within bags (3 per bag, 96 in total) were modelled at the residual level.

The experimental layout followed a split-plot design without formal blocking, as trees were randomly distributed throughout the orchard. In this design, the maternal parent (applied to a tree) constituted the main plot factor, while the paternal parent (pollen donor applied to a bag) represented the split-plot factor. Each bag serves as a split plot within the tree (main plot) (Table 6). In the first (tree) stratum, the cross includes the maternal effect and is associated with 3 denominator degrees of freedom. The second (bag within tree) stratum partitions the cross into paternal effects and the interaction between maternal and paternal effects, resulting in 12 denominator degrees of freedom. The appropriate denominator degrees of freedom for the overall cross are calculated as a weighted average of the residual degrees of freedom from both strata. Given that the residual from the second stratum carries greater weight, the resulting denominator degrees of freedom for the cross is a non-integer value (supplementary table S1).

**Table 6:** Summary of the split-plot experimental design, detailing the plot and treatment structure components along with their corresponding degrees of freedom (df)

Source (plot structure)	Numerator df	Source (treatment structure)	Denonimator df
Mean	1	Mean	1
Tree	7	Cross Mother	3 3
		Residual	**4**
Tree: Bag	24	Cross Father Mother: Father	12 3 9
		Residual	**12**
Tree: Bag: Raceme (residual)	64		

**Fig. 10:**
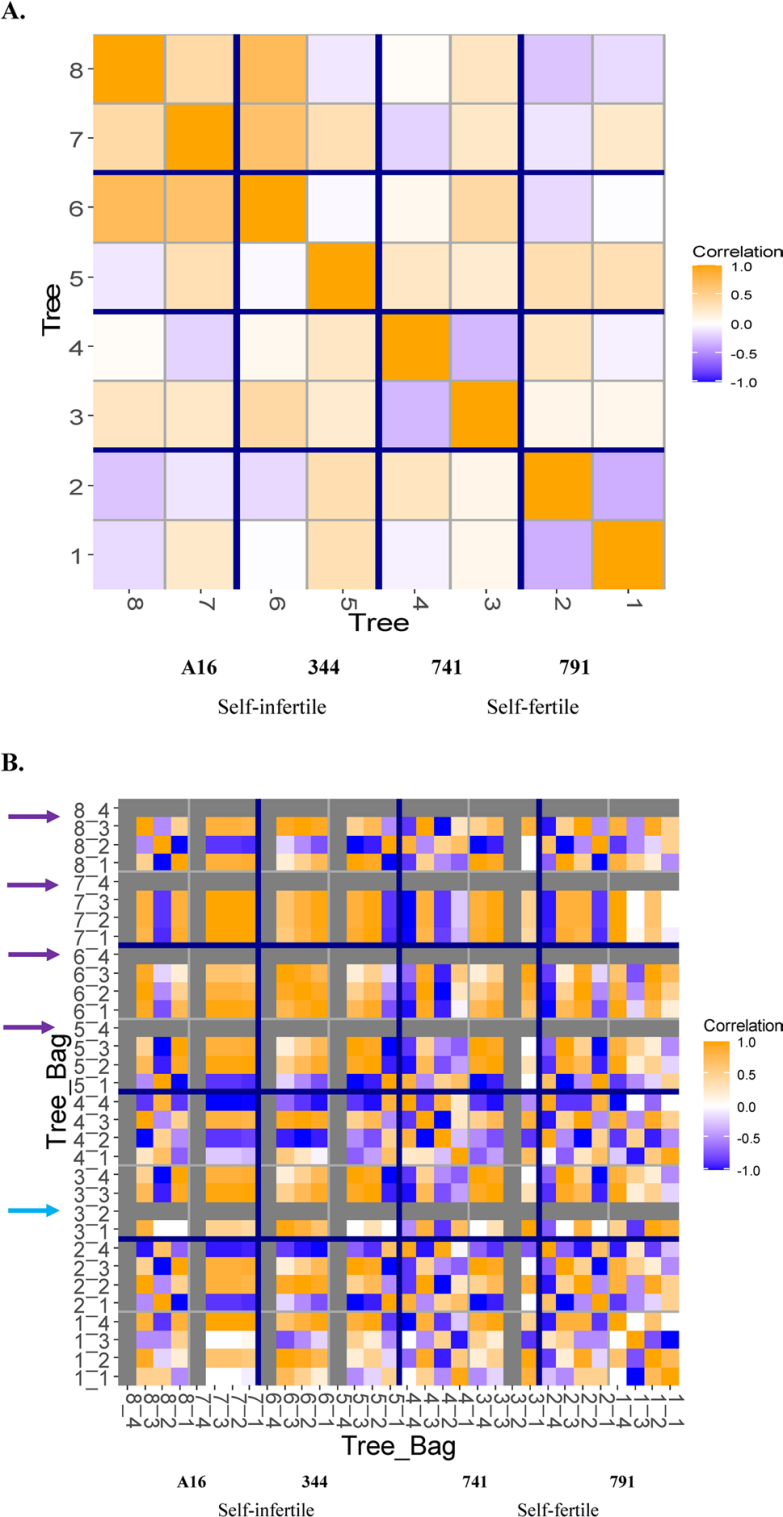
Heat map illustrating correlation among plot structure terms, grouped by self-fertile (‘791’, ‘741’) and self-infertile (‘344’, ‘A16’) cultivars. Trees from self-fertile (‘791’, ‘741’) and self-infertile (‘344’, ‘A16’) group. **A** Correlation between trees across four cultivars, with two trees per cultivar. Dark blue lines delineate cultivar boundaries, and dark grey lines delineate individual trees. Positive correlations are observed among self-infertile trees, while negative correlations are evident among self-fertile trees. **B** Correlation between Tree: Bag combinations, with four bags per tree. Dark blue lines delineate cultivars, and dark grey lines delineate trees. Self-infertile self-pollinations (‘344 × 344’ and ‘A16 × A16’; marked with purple arrows) and the cross ‘741 × 344’ (blue arrow) show no detectable correlation

Following model execution, it was observed that the plot structure terms— tree and bag nested within tree, were constrained to zero. To enable accurate estimation of fixed effects, these plot structure terms were unconstrained, resulting in negative variance component estimates for these terms (supplementary table S3). The presence of negative variance components suggests that variability among main plot units (trees) is lower than the variability contributed by their subunits (bags).This would happen if the subunits were negatively correlated (Nelder 1954; Payne et al. 2011). In our study, substantial variation in correlation was observed among trees of the same cultivar (especially ‘741’ and ‘791’) while the correlation between trees from different cultivars were more variable (Fig. 10A). Additionally, the correlation within bags was greater than between bags (Fig. 10B), potentially due to incomplete pollination of florets by the intended pollen donors. The correlation range between bags on different trees of the same cultivar (e.g., ‘741’ and ‘791’) was larger compared to that between bags from different cultivars (Fig. 10B). Notably, self-infertile crosses such as ‘344 × 344’ and ‘A16 × A16’, as well as the cross ‘741 × 344’, exhibited no correlation, consistent with the absence of pollen tube growth reaching the lower style in their pistils (refer to the Results section for further details). Collectively, these patterns contribute to the estimation of negative variance components in the plot structure terms, as described in (Nelder 1954).

## Data Availability

Data will be available upon request. Please contact [m.alam@uq.edu.au](mailto: m.alam@uq.edu.au).
